# Disruption of the *Trichoderma reesei gul1* gene stimulates hyphal branching and reduces broth viscosity in cellulase production

**DOI:** 10.1093/jimb/kuab012

**Published:** 2021-02-10

**Authors:** Qinqin Zhao, Qin Liu, Qi Wang, Yuqi Qin, Yaohua Zhong, Liwei Gao, Guodong Liu, Yinbo Qu

**Affiliations:** State Key Laboratory of Microbial Technology, Shandong University, 72 Binhai Road, 266237 Qingdao, China; State Key Laboratory of Microbial Technology, Shandong University, 72 Binhai Road, 266237 Qingdao, China; National Glycoengineering Research Center, Shandong University, 27 Binhai Road, 266237 Qingdao, China; National Glycoengineering Research Center, Shandong University, 27 Binhai Road, 266237 Qingdao, China; State Key Laboratory of Microbial Technology, Shandong University, 72 Binhai Road, 266237 Qingdao, China; State Key Laboratory of Microbial Technology, Shandong University, 72 Binhai Road, 266237 Qingdao, China; Tobacco Research Institute of Chinese Academy of Agricultural Sciences, 11 Keyuanjingsi Road, 266101 Qingdao, China; State Key Laboratory of Microbial Technology, Shandong University, 72 Binhai Road, 266237 Qingdao, China; National Glycoengineering Research Center, Shandong University, 27 Binhai Road, 266237 Qingdao, China; State Key Laboratory of Microbial Technology, Shandong University, 72 Binhai Road, 266237 Qingdao, China; National Glycoengineering Research Center, Shandong University, 27 Binhai Road, 266237 Qingdao, China

**Keywords:** Hyphal morphology, *Trichoderma reesei*, Cellulase, Protein production, Filamentous fungus

## Abstract

Hyphal morphology is considered to have a close relationship with the production level of secreted proteins by filamentous fungi. In this study, the *gul1* gene, which encodes a putative mRNA-binding protein, was disrupted in cellulase-producing fungus *Trichoderma reesei*. The hyphae of Δ*gul1* strain produced more lateral branches than the parent strain. Under the condition for cellulase production, disruption of *gul1* resulted in smaller mycelial clumps and significantly lower viscosity of fermentation broth. In addition, cellulase production was improved by 22% relative to the parent strain. Transcriptome analysis revealed that a set of genes encoding cell wall remodeling enzymes as well as hydrophobins were differentially expressed in the Δ*gul1* strain. The results suggest that the regulatory role of *gul1* in cell morphogenesis is likely conserved in filamentous fungi. To our knowledge, this is the first report on the engineering of *gul1* in an industrially important fungus.

## Introduction

The ascomycete fungus *Trichoderma reesei* (teleomorph *Hypocrea jecorina*) is widely used for industrial cellulase production in the world (Bischof et al., 2016). The cellulase hyper-producing mutant of *T. reesei* was reported to produce up to 100 g/l of proteins in industry (Cherry & Fidantsef, 2003). Moreover, *T. reesei* has been developed as a promising chassis for the production of heterologous proteins (e.g., lipase and pharmaceutical proteins) (Landowski et al., 2016; Rantasalo et al., 2019). Therefore, understanding the biological processes involved in protein production in *T. reesei* is important for rational engineering of strains for industrial applications. In the past decades, most of the work in this field has been focused on transcriptional regulation, which critically affects the synthesis level of cellulases (Druzhinina & Kubicek, 2017).

The linkage between morphology and productivity has been observed in many industrial filamentous fungi (Grimm et al., 2005; Quintanilla et al., 2015). First, the filamentous morphology of cells affects the viscosity and consequently the efficiency of mass transfer in the fermentation broth. Second, the action of shear stress on long hyphae could be harmful to cell health in submerged fermentation. Third, the frequency of hyphal branching might have a direct influence on secreted protein production, as protein secretion is reported to be preferably occur at hyphal tips (Li et al., 2019; Wosten et al., 1991). In *T. reesei*, freely dispersed or clumped mycelia are generally observed during submerged fermentation for cellulase production (Ahamed & Vermette, 2009; Choy et al., 2011). Using different cultivation media, a positive correlation was observed between the number of tips and cellulase production (Ahamed & Vermette, 2009). Hyphal pellets were also reported for *T. reesei* cultures at low inoculum size or in the presence of specific kinds of surfactants (Callow & Ju, 2012; Domingues et al., 2000). Because the changes of cultivation parameters often affect multiple cell functions (e.g., nutrition and membrane permeability), the relationship between morphology and cellulase production is hard to discern in many studies.

Genetic engineering of morphology has been performed in some fungal species based on the knowledge of cell growth and development. For example, deletion of a kinesin-encoding gene *kipA* in *Aspergillus glaucus* resulted in more compact mycelial clumps, lower viscosity of culture, and higher production of aspergiolide A than the parent strain (Cai et al., 2014). Through the screening of 90 gene deletion mutants with morphological changes, Lin et al. (2018) found that the disruption of *gul-1* gene in *Neurospora crassa* caused the formation of hyphal pellets in submerged cultivation, which significantly reduced the viscosity of culture. The protein product of *gul-1* and nuclear DBF2-related (NDR) kinase COT-1 comprise a pathway regulating cell wall integrity and morphogenesis in *N. crassa* (Herold & Yarden, 2017; Terenzi & Reissig, 1967). This pathway has been studied more comprehensively in *Saccharomyces cerevisiae*, where the activity of Ssd1 (GUL-1 homolog) is regulated by CBK1 (COT-1 homolog) through phosphorylation (Kurischko, Kim, et al., 2011). During polarized growth, phosphorylated Ssd1 binds to a specific set of mRNAs and promotes their asymmetric localization. Under stress conditions, Ssd1 is suggested to be dephosphorylated, and carries the mRNAs binds to mRNA processing bodies (P-bodies) and stress granules to repress their translation (Kurischko, Kim, et al., 2011; Kurischko, Kuravi, et al., 2011).

While the close homologs of GUL-1/Ssd1 are present in many industrial fungi, their roles in morphogenesis and the relevant strain engineering have been less reported. In this study, we performed the disruption of *gul1* gene in *T. reesei*. The *gul1* disruption mutant exhibited a hyper-branching morphology and reduced cell wall integrity compared with the parent strain. In addition, the mutant showed a lower viscosity of fermentation broth and higher cellulase production than the parent, suggesting that *gul1* disruption is an effective strategy for morphological engineering of *T. reesei*.

## Materials and Methods

### Construction of Strains

*T. reesei* QP4, a uracil auxotrophic strain derived from the strain QM9414 through deleting the *pyr4* gene (Zhong et al., 2016), was used as a parent for strain construction. The *gul1* gene knockout cassette was constructed using the double-joint PCR method (Yu et al., 2004). First, the upstream and downstream sequences of *gul1* were amplified from the genomic DNA of QP4 using primer pairs gul1-UF/gul1-UR and gul1-DF/gul1-DR, respectively. The *Aspergillus niger pyrG* gene was amplified from the genomic DNA of *T. reesei* SCB18 using primer pair pyrG-F1/pyrG-R1, and employed as a selection marker. The SCB18 strain carries *A. niger pyrG* gene after previously reported genetic manipulations (Gao, Qian, et al., 2017). The above fragments were fused together, and the primer pair gul1-NF/gul1-NR was used as nested primers to amplify the entire gene knockout cassette. The *gul1* overexpression cassette was constructed by fusing the P*pdc1* promoter, *gul1* coding and downstream sequences, and *A. niger pyrG* together. The cassettes were then transformed into the protoplasts of QP4 as described by Penttilä et al. (1987). Transformants were screened and purified on minimal medium plates, and identified by PCR using indicated primers. To construct the reference strain QPP, the *A. niger pyrG* gene was transformed into the QP4 protoplasts, and the transformants with *pyrG* integrated into the genome were identified through PCR using the primer pair pyrG-F1/pyrG-R1. All the primers used in this study were listed in Supplementary Table S1.

### Cultivation

The strains were cultivated on potato dextrose agar (PDA) plates at 30°C for 7 days for conidiation. The conidia were harvested by washing PDA plates with distilled water containing 0.9% (wt/vol) NaCl and 0.01% (wt/vol) Tween 80. For mycelial growth study, fresh conidia were inoculated into 50 ml minimal medium at a final concentration of 10^6^ per ml, and the Erlenmeyer flasks were incubated in a rotary shaker at 200 rpm at 30°C. For cellulase production, the strains were first grown in minimal medium for 36 hr, and then 5 ml culture was inoculated to 50 ml cellulase production medium for continued cultivation.

The minimal medium contained (g/l): glucose 20.0, (NH_4_)_2_SO_4_ 5.0, KH_2_PO_4_ 15.0, MgSO_4_·7H_2_O 0.6, CaCl_2_ 0.6, peptone 2.0, FeSO_4_·7H_2_O 0.005, MnSO_4_·H_2_O 0.0016, ZnSO_4_·7H_2_O 0.0014, and CoCl·6H_2_O 0.002. The cellulase production medium contained (g/l): microcrystalline cellulose 20.0, corn steep liquor 20.0, KH_2_PO_4_ 5.0, (NH_4_)_2_SO_4_ 2.0, MgSO_4_·7H_2_O 0.6, and CaCl_2_ 1.0.

### Phenotype Analysis on Agar Plates

One microliter of conidial suspension (10^6^ per ml) of strains was inoculated on the center of PDA or cellulose agar plates, and then cultivated at 30°C. The cellulose agar plate was the same with minimal medium agar plate except that glucose was replaced by 2% (wt/vol) ball-milled cellulose. The diameters of colonies on agar plates were measured every day. For stress sensitivity analysis, the strains were inoculated on minimal medium plates supplied with different chemicals as indicated, and cultivated at 30°C unless specifically stated.

### Microscopy Analysis

The conidia were inoculated to cellulose agar plates with coverslips inserted to the medium. Images of hyphae on cellulose agar plates or mycelia in liquid minimal medium were acquired with Eclipse 80i upright microscope (Nikon, Japan), and analyzed using the ImageJ 1.8.0 software (Schneider et al., 2012). The *L*_hgu_ (length of a hyphal growth unit) value was calculated by dividing the hyphal length by the number of tips (Quintanilla et al., 2015). Fifty hyphae were measured for each strain.

### Biomass Measurement

The mycelial biomass in 50 ml liquid minimal medium was collected by vacuum filtration and washed with distilled water. The mycelia were dried to constant weight at 60°C and weighed. Due to the insolubility of microcrystalline cellulose, the biomass in cellulase production medium was measured indirectly by determining the amount of internal protein. Specifically, 1 ml of culture broth was centrifuged at 8,000 *g* for 30 min, and then the precipitate was washed with 0.9% (wt/vol) NaCl solution. Next, the precipitate was resuspended in 1 ml of 1 M NaOH solution and incubated at 200 rpm for 1 h at room temperature. The suspension was centrifuged at 8,000 *g* for 10 min, and the protein content of the supernatant was determined by the Bradford Protein Assay Kit (Sangon Biotech, Shanghai, China).

### Viscosity Measurement

The viscosity of fermentation broth was measured using digital viscometer NDJ-5S (Lichen Bangxi Instrument Co. Ltd., Shanghai, China) according to the manufacturer's instructions. The instrument drives a spindle (immersed in the test sample) through a calibrated spring, and measures the viscous drag of the fluid against the spindle by the spring deflection. One hundred ml of culture broth in a 100 ml beaker was used for measurement with three repeats at room temperature. No. 2 spindle supplied with the viscometer was used with the rotation speed set at 6 rpm. Data in the measurement range of 20– 40% were recorded after the readings were stable.

### Cellulase Activity Assay and SDS–PAGE

The culture broth was centrifuged at 8,000 *g*, 4°C for 10 min to collect supernatant. Filter paper activity was determined with Whatman No. 1 filter paper as the substrate as previously described (Gao, Li, et al., 2017). One unit of enzyme activity was defined as the amount of enzyme required to release 1 μmol glucose equivalent from the substrate per minute. For SDS–PAGE, equal volumes (15 µl) of culture supernatants were supplemented with loading buffer, boiled for 10 min, and loaded onto a 12% SDS polyacrylamide separating gel for electrophoresis at 120 V for 1 hr.

### RNA-seq

The strains were first grown in minimal medium for 36 hr. Then, 5 ml culture was inoculated to 50 ml cellulase production medium with 10 g/l microcrystalline cellulose as the sole carbon source, and then cultured for 48 hr in biological triplicates. Mycelia were harvested by vacuum filtration and frozen immediately in liquid nitrogen. Total RNA was isolated from ground mycelia using RNAiso Reagent (TaKaRa, Japan) according to the manufacturer's instructions. High-throughput sequencing of RNA samples was performed by Personal Biotechnology Co., Ltd. (Shanghai, China). Briefly, mRNA was purified from total RNA using poly-T oligo attached magnetic beads, and then sequencing libraries were generated using the TruSeq RNA Sample Preparation Kit (Illumina, San Diego, CA, USA). The products with an average insert size of 380 bp were purified using the AMPure XP system (Beckman Coulter, Beverly, CA, USA), and quantified using the Agilent high sensitivity DNA assay on a Bioanalyzer 2100 system (Agilent). Paired-end sequencing was performed on a NovaSeq 6000 platform (Illumina) with a read length of 150 bp. The clean reads obtained after raw data processing were mapped to the reference genome of *T. reesei* QM6a (NCBI assembly accession: GCF_000167675.1) using HISAT2 (Kim et al., 2019). Count values on each gene were quantified using HTSeq (Anders et al., 2015). FPKM (fragments per kilobase per million mapped fragments) was used to standardize the gene expression values. DESeq 1.30.0 (Anders & Huber, 2010) was used to identify the genes of significantly differential expression with combined thresholds (|log2FoldChange| > 1 and *p* < .05).

### Prediction of GPI Anchored Proteins

The list of proteins with signal peptides predicted by SignalP was downloaded from the JGI genome portal (https://mycocosm.jgi.doe.gov/Trire2/Trire2.home.html). The proteins were then used for the prediction of GPI anchored proteins using the online tool NetGPI-1.0 (Gíslason et al., 2021).

### Statistical Analysis

Statistical significance tests of differences were performed by calculating *p* values with two-tailed homoscedastic *t*-test in the software Microsoft Office 2016 Excel (Microsoft, USA).

## Results

### Annotation and Disruption of the *gul1* Gene in *T. reesei*

Reciprocal BLASTp analysis identified the protein product of gene Trire2_77084 (NCBI RefSeq accession number: XP_006964501.1) in *T. reesei* wild-type strain QM6a as the ortholog of *N. crassa* GUL-1 (Martinez et al., 2008). However, the predicted protein sequence of *T. reesei* GUL1 lacks around 250 amino acids at the N-terminal when aligned with *N. crassa* GUL-1. Manual check of the genome sequence suggested that the *gul1* gene was mis-annotated in strain QM6a, while the annotation in mutant strain Rut-C30 (TrireRUTC30_1_24555; GenBank accession number: ETS03006.1) is correct.

The 1,349 amino acid-long sequence of *T. reesei* GUL1 has an identity of 76.8% with *N. crassa* GUL-1. Although predicted to have a ribonuclease II/R domain, *T. reesei* GUL1 should not have a ribonuclease activity due to the lack of some key amino acid residues (e.g., metal ion binding sites) for catalysis, which is a common feature for GUL-1/Ssd1 orthologs (Supplementary Fig. S1). In addition, *T. reesei* GUL1 possesses several putative Cbk1/COT-1 phosporylation sites, of which some are conserved between *S. cerevisiae, N. crassa*, and *T. reesei* (data not shown).

The major part of *gul1* gene was deleted via homologous recombination in *T. reesei* QP4, a uridine auxotrophic strain constructed from strain QM9414 (Zhong et al., 2016) (Supplementary Fig. S2). One of the generated *gul1*-disrupted mutants was named Δ*gul1* and used for further study. To exclude possible differences between genetic complementation and nutritional supplementation (Pronk, 2002), QP4 was also transformed with the selection marker gene *pyrG* to generate the auxotrophy-complemented reference strain QPP (via random integration). In this study, there is actually no significant difference between QP4 and QPP when uracil was supplemented in the culture medium.

### The Disruption of *gul1* Increased Hyphal Branching

**Fig. 1.**
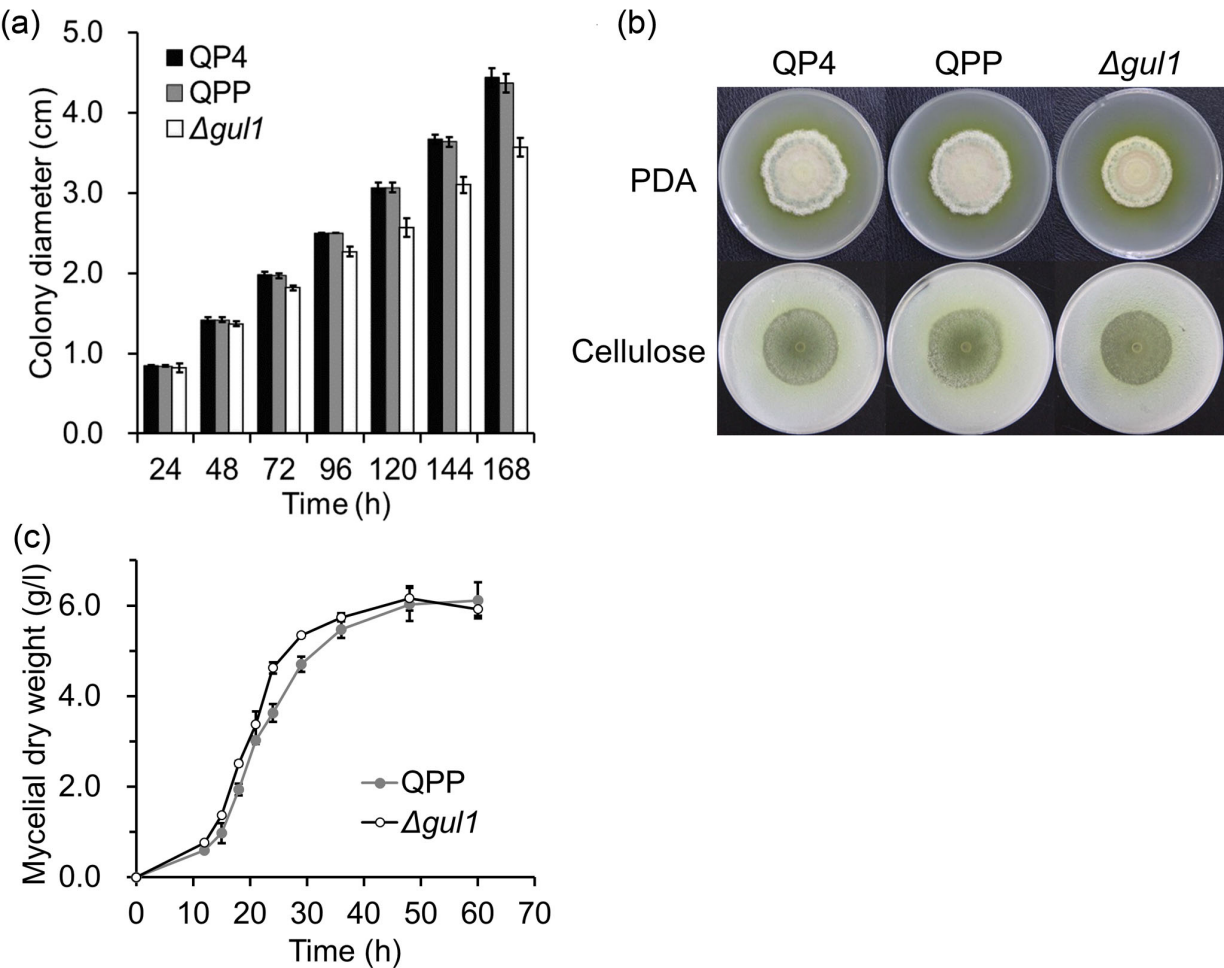
The effect of *gul1* disruption on the growth of *T. reesei*. For the growth on agar plates, Triton X-100 at a concentration of 0.1% (wt/vol) was included in the media. (a) The diameters of fungal colonies on PDA plates. Data represent mean ± S.D. (error bars) from triplicate cultivations. (b) The morphology of colonies on PDA plates and cellulose agar plates (see section Materials and Methods). Photos were taken after 168 hr of cultivation. (c) The growth in liquid minimal medium. Data represent mean ± S.D. (error bars) from triplicate cultivations.

On PDA plate, the Δ*gul1* strain showed a slower radial growth than reference strains QP4 and QPP (Fig. 1a). In addition, the colony of Δ*gul1* was more compact, and had a smoother edge, than those of reference strains (Fig. 1b). On the medium with cellulose as the sole carbon source, the difference in colony morphology among strains was less remarkable.

The growth of strains was also compared in liquid medium with 2% (wt/vol) glucose as the sole carbon source. The Δ*gul1* strain accumulated higher biomass than the reference strain QPP at the early stage of cultivation (Fig. 1c). Nevertheless, the calculated maximum specific growth rate of Δ*gul1* (0.15 h^−1^) was lower than that of QPP (0.19 h^−1^). This result was consistent with the higher germination rate of the conidia of Δ*gul1*. According to microscopic observation, the mean percent of germination for conidia of Δ*gul1* was 24.84% (54/213) after 10 hr of incubation, while that of QP4 and QPP was 14.00% (35/250) and 13.81% (25/181), respectively.

**Fig. 2.**
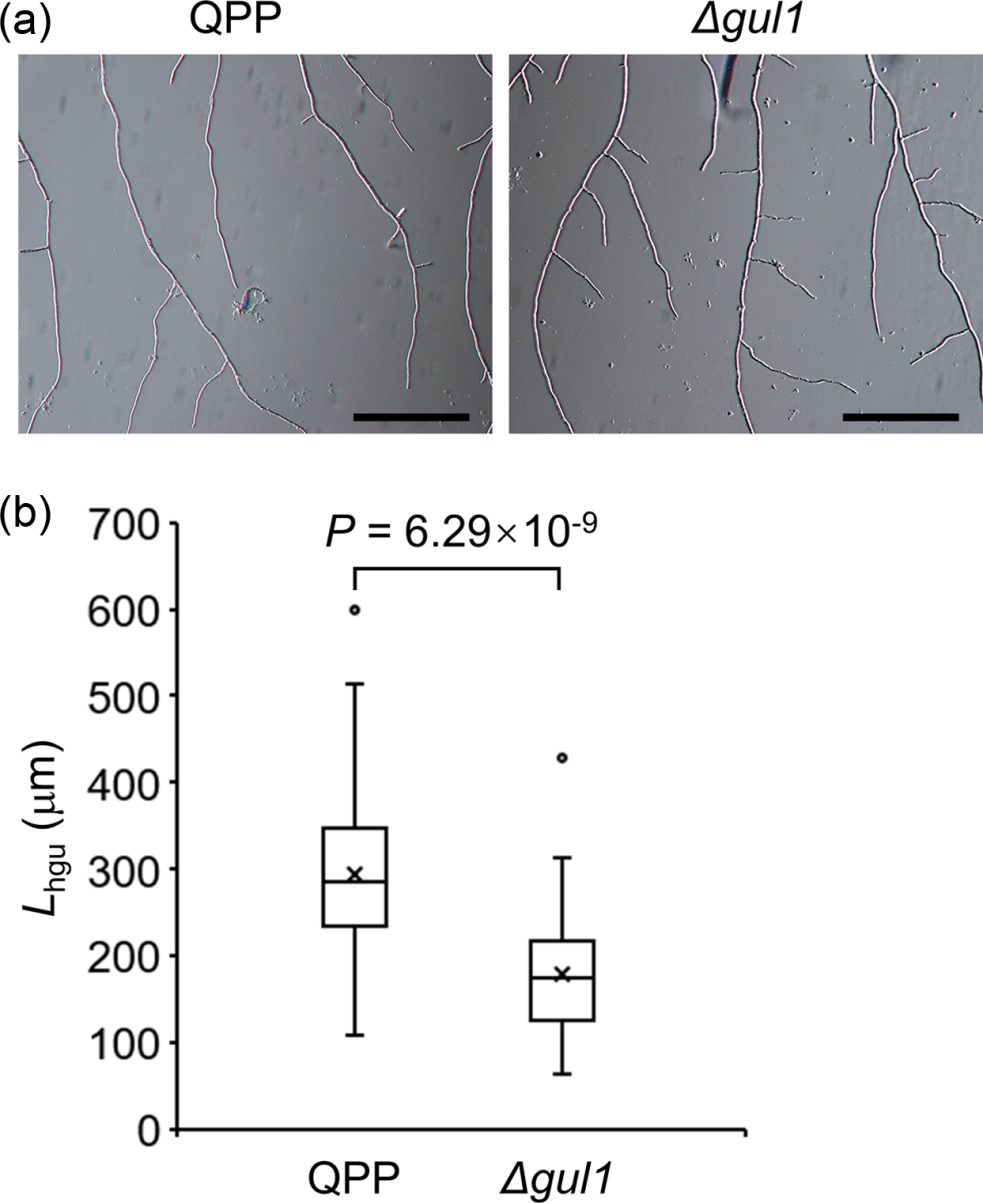
Disruption of *gul1* increased hyphal branching on cellulose agar plates. Photos were taken after 24 hr of cultivation. (a) The morphology of hyphae. Scale bar, 50 μm. (b) The *L*_hgu_ values (see section Materials and Methods) quantified by measuring 50 hyphae for each strain. The cross markers indicate mean values.

The Δ*gul1* strain showed a hyper lateral branching phenotype on cellulose agar plate when examined with microscope (Fig. 2a). The significant lower *L*_hgu_ value of Δ*gul1* than QPP (179.6 μm versus 293.5 μm) clearly suggested that *gul1* disruption increased hyphal branching (Fig. 2b). This hyper-branching phenotype was in line with the lower radial growth rate of Δ*gul1*.

### The *Δgul1* Strain Formed Smaller Clumps in Liquid Medium

**Fig. 3.**
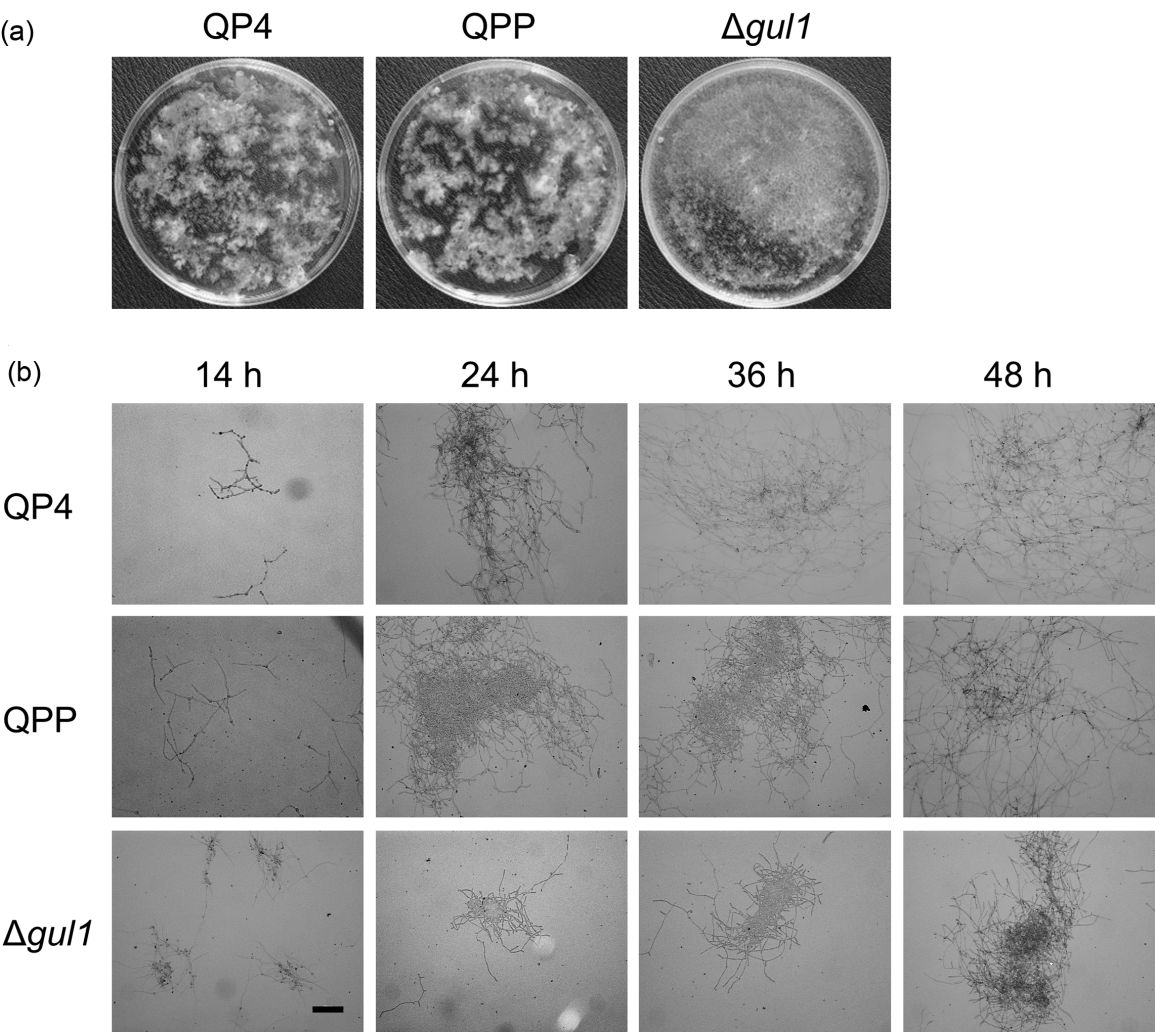
The effect of *gul1* disruption on the morphology in liquid minimal medium. (a) The macro-morphology of strains in shake flasks. The culture broths after 36 hr of cultivation were poured into 9-cm petri dishes for photo taking. (b) Representative microscopic images of mycelia at different time points of cultivation. Scale bar, 100 μm.

The disruption of *gul1* also changed the morphology of mycelia in liquid medium. The mycelia of reference strains were wound into clumps in liquid minimal medium, with irregular shapes and uneven distributions. By contrast, the mycelial clumps of Δ*gul1* were more uniform and inclined to be pelleted (Fig. 3a). The different morphologies of mycelia were also remarkable when examined under microscope. While the hyphae of reference strains were intertwined into network structures, Δ*gul1* formed smaller and more compact clumps throughout the cultivation (Fig. 3b).

### The Δ*gul1* Strain Showed Lower Culture Viscosity and Increased Protein Production During Cellulase Fermentation

**Fig. 4.**
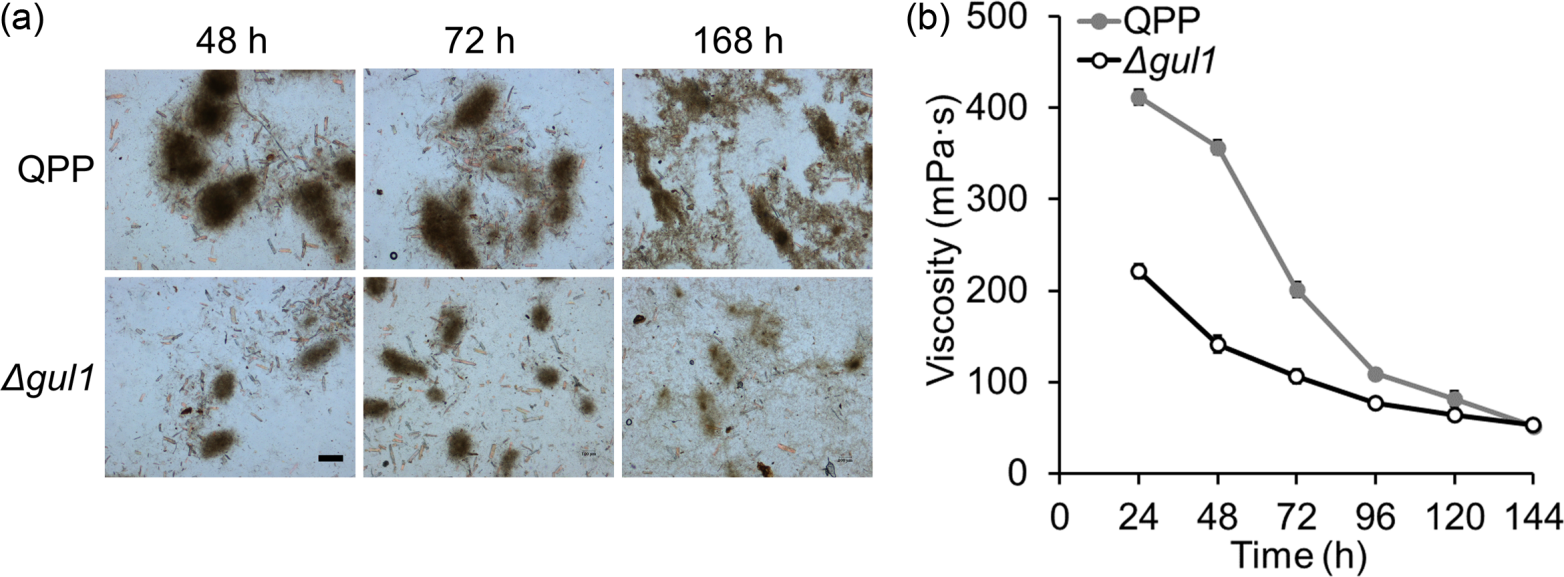
Comparison of the morphology and broth viscosity of strains during cellulase production. (a) Representative microscopic images of mycelia. Scale bar, 200 μm. (b) The viscosities of fermentation broths. Data represent mean ± S.D. (error bars) from duplicate cultivations.

Considering the wide use of *T. reesei* in cellulase production, we compared the Δ*gul1* and reference strains in a cellulase production medium, where 2% (wt/vol) cellulose served as a carbon source. Under this condition, the strains formed more compact mycelial clumps relative to those in glucose medium. Similarly, smaller mycelial clumps were observed for Δ*gul1* than the reference strains (Fig. 4a). In addition, the broth viscosity of Δ*gul1* was dramatically lower than that of the reference strain, particularly in the early stage of cultivation (Fig. 4a). The viscosity at 48 hr was 141.3 mPa s for Δ*gul1*, which was approximately 40% of that of reference strain QPP (356.7 mPa s). Mycelial autolysis was observed after 120 hr, when the broth viscosity dropped below 100 mPa s for both strains.

**Fig. 5.**
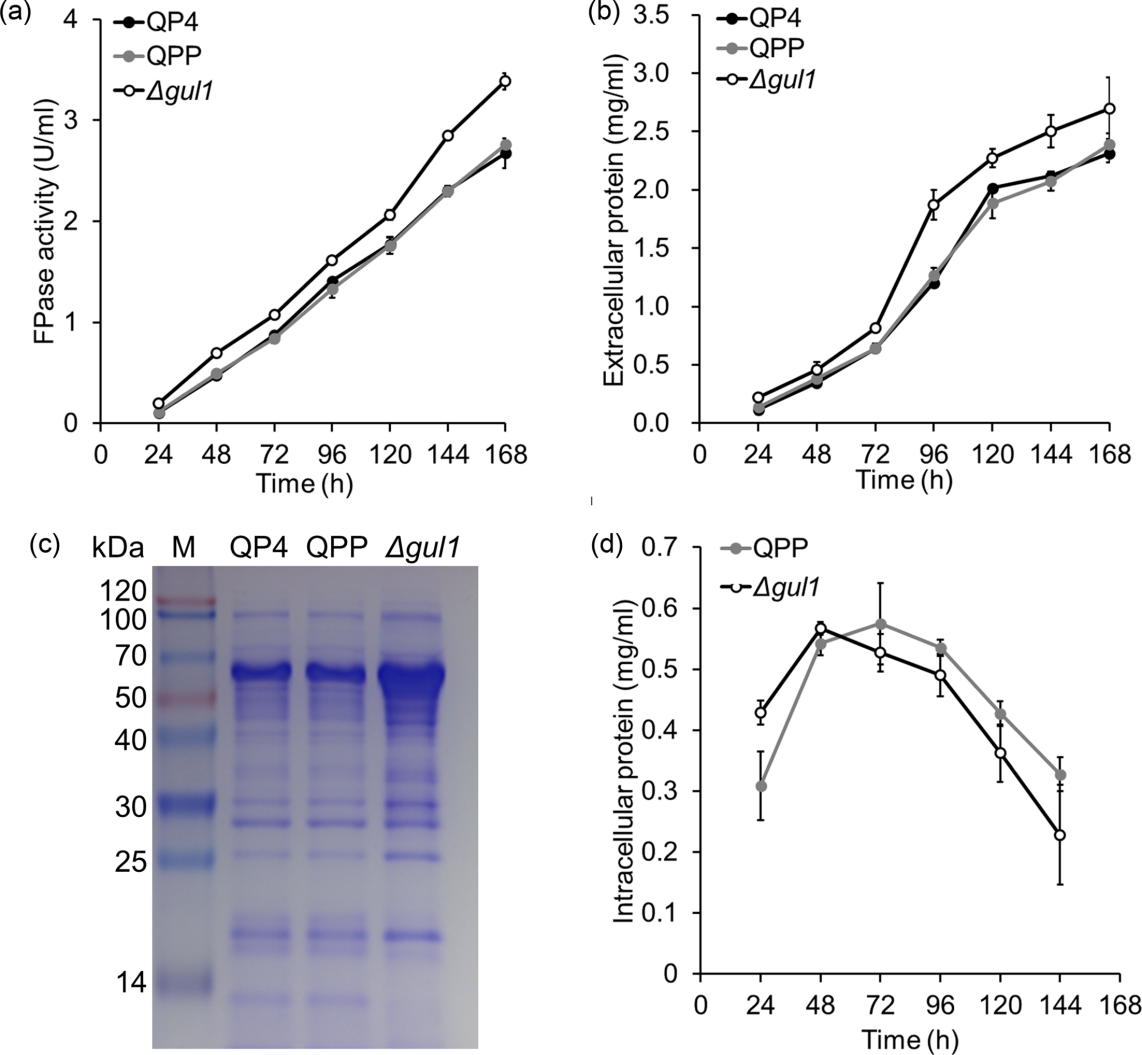
Cellulase production by Δ*gul1* and reference strains. (a) FPase activity. (b) Extracellular protein concentration. (c) SDS–PAGE of culture supernatants sampled at 96 hr. The samples were loaded with equal volumes (30 μl). (d) Intracellular protein concentration representing biomass level. Data represent mean ± S.D. (error bars) from triplicate cultivations.

The production level of cellulase (measured as filter paper enzyme, FPase) of the Δ*gul1* strain was higher than those of reference strains (Fig. 5a). The cellulase activity of Δ*gul1* at 168 hr was 3.38 U/ml, 22% higher than that of QPP. Consistently, Δ*gul1* produced more proteins to the culture, particularly for some proteins with apparent molecular weights of ∼60 kDa (Fig. 5b and c). The production level of cellulase was similar with those reported for *T. reesei* in many studies, such as 1.9 U/ml (Culbertson et al., 2013), 2.0–4.5 U/ml (Gao, Qian, et al., 2017a), and 1.6–2.3 U/ml (Novy et al., 2016). Nevertheless, further engineering of genes directly involved in cellulase production (e.g., transcription factors) and process optimization are needed to achieve higher production levels (Ellilä et al., 2017; Novy et al., 2019).

To clarify if the decrease broth viscosity and increased protein production in Δ*gul1* was due to any change in cell biomass abundance, intracellular proteins were extracted and determined to indirectly study cell growth in cellulose medium (Bischof et al., 2013). As shown in Fig. 5d, the growth of Δ*gul1* was in advance compared with the reference strain, with a higher biomass accumulated before 48 hr. The maximum biomass was similar between the strains. Thus, the decreased viscosity of fermentation broth for Δ*gul1* should be due to the change in morphology of mycelium.

**Fig. 6.**
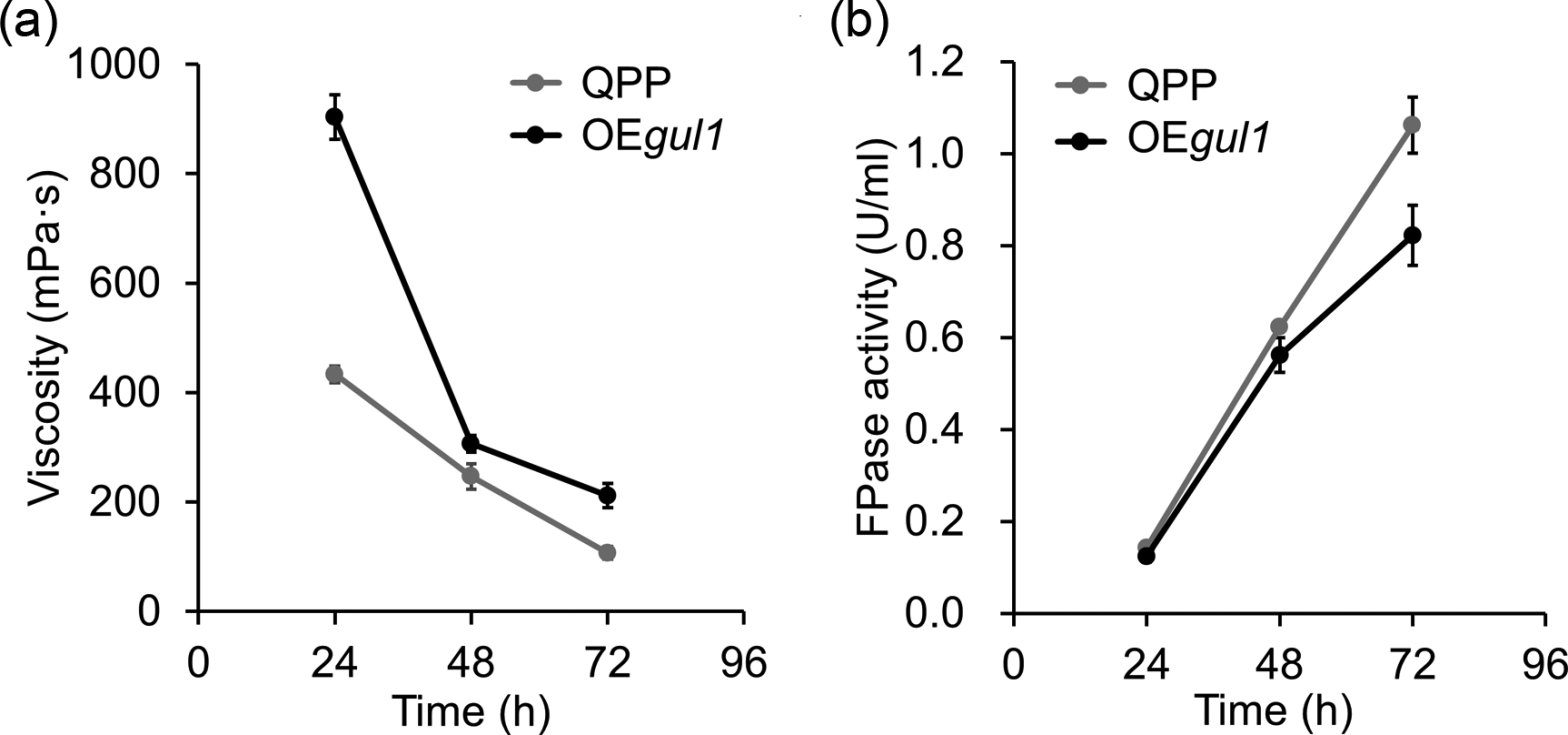
The effect of *gul1* overexpression on cellulase production. (a) The viscosities of fermentation broths of OE*gul1* and reference strain QPP. (b) Cellulase production by OE*gul1* and QPP. Data represent mean ± S.D. (error bars) from triplicate cultivations.

The relationship between *gul1* and broth viscosity was further confirmed by the construction and examination of the *gul1*-overexpression strain OE*gul1* (Supplementary Fig. S2). In the cellulase production medium, the broth of OE*gul1* showed significantly higher viscosities than that of QPP, especially in the early stage of cultivation (Fig. 6a). The cellulase production level of OE*gul1* was similar with that of QPP at 24 hr, but became lower along with the fermentation (Fig. 6a). In summary, the results of *gul1* overexpression study are in agree with those from *gul1* disruption.

### Disruption of *gul1* Caused a Modest Transcriptomic Change During Cellulase Production

While Ssd1 is believed to mainly regulate the translation of its target mRNAs, it could also stabilize at least some of the targets and therefore affect the abundance of transcripts (Ohyama et al., 2010). In addition, the changes in mycelial morphology and cell wall integrity (see next section) caused by *gul1* disruption may indirectly affect the transcription level of some genes. Therefore, the transcriptomes of Δ*gul1* and QPP grown in cellulose medium (at 48 hr) were compared using the RNA-seq technology.

Among the 9,113 genes analyzed, 272 and 249 genes were significantly upregulated and downregulated, respectively, in Δ*gul1* relative to QPP (Supplementary Table S2 and Supplementary Fig. S3). Gene Ontology term enrichment of the differentially expressed genes suggested that the transcript abundance of membrane proteins, glycoside hydrolases, and oxidoreductases had more significant changes. Within the 228 glycoside hydrolase, carbohydrate esterase and polysaccharide lyase genes annotated by Häkkinen et al. (2012), 17 genes were significantly upregulated, while 42 were downregulated, in Δ*gul1* (Supplementary Table S2). These genes included 6 of the 18 chitinase genes in *T. reesei* (Seidl et al., 2005). Specifically, *chi18-12* and *chi18-15* were upregulated, while *chi18-14, chi18-16, chi18-17*, and *chi18-18* were downregulated, in *Δgul1* (Supplementary Fig. S3). In addition, genes encoding chitosanases, α-1,6-mannanases and β-1,3-glucanases, which are probably involved in cell wall remodeling, were also found in the differentially expressed genes.

Glycosylphosphatidylinisotol (GPI) anchored proteins, which are known to be attached to membrane or cell wall in fungi, are involved in cell wall biogenesis, integrity and cell adhesion (Gonzalez et al., 2009). Eighty-six GPI anchored protein-encoding genes were predicted in *T. reesei* (see section Materials and Methods), of which 14 were differentially expressed in in Δ*gul1* relative to QPP. These genes are predicted to encode a β-1,3-glucanosyltransferase, an α-1,6-mannanase, and putative cell wall mannoproteins with unknown functions (Supplementary Table S3). The significant upregulation of two hydrophobin genes was also noted. The gene *hfb1* with a role in hyphal development (Askolin et al., 2005) and another class II hydrophobin gene TRIREDRAFT_106538 were upregulated by 9.91- and 10.83-fold, respectively, in Δ*gul1* relative to QPP.

Unexpectedly, the transcription levels of genes encoding major cellulases and hemicellulases were decreased in Δ*gul1* (Supplementary Table S2). In addition, the gene *xyr1*, which encodes a key transcriptional activator for cellulase/hemicellulase expression (Stricker et al., 2006), was downregulated (by around 40%) in Δ*gul1*. These results suggested that the improved cellulase production in Δ*gul1* strain was not likely due to increased cellulase gene expression.

### The *gul1* Gene is Involved in Cell Wall Integrity Maintenance in *T. reesei*

**Fig. 7.**
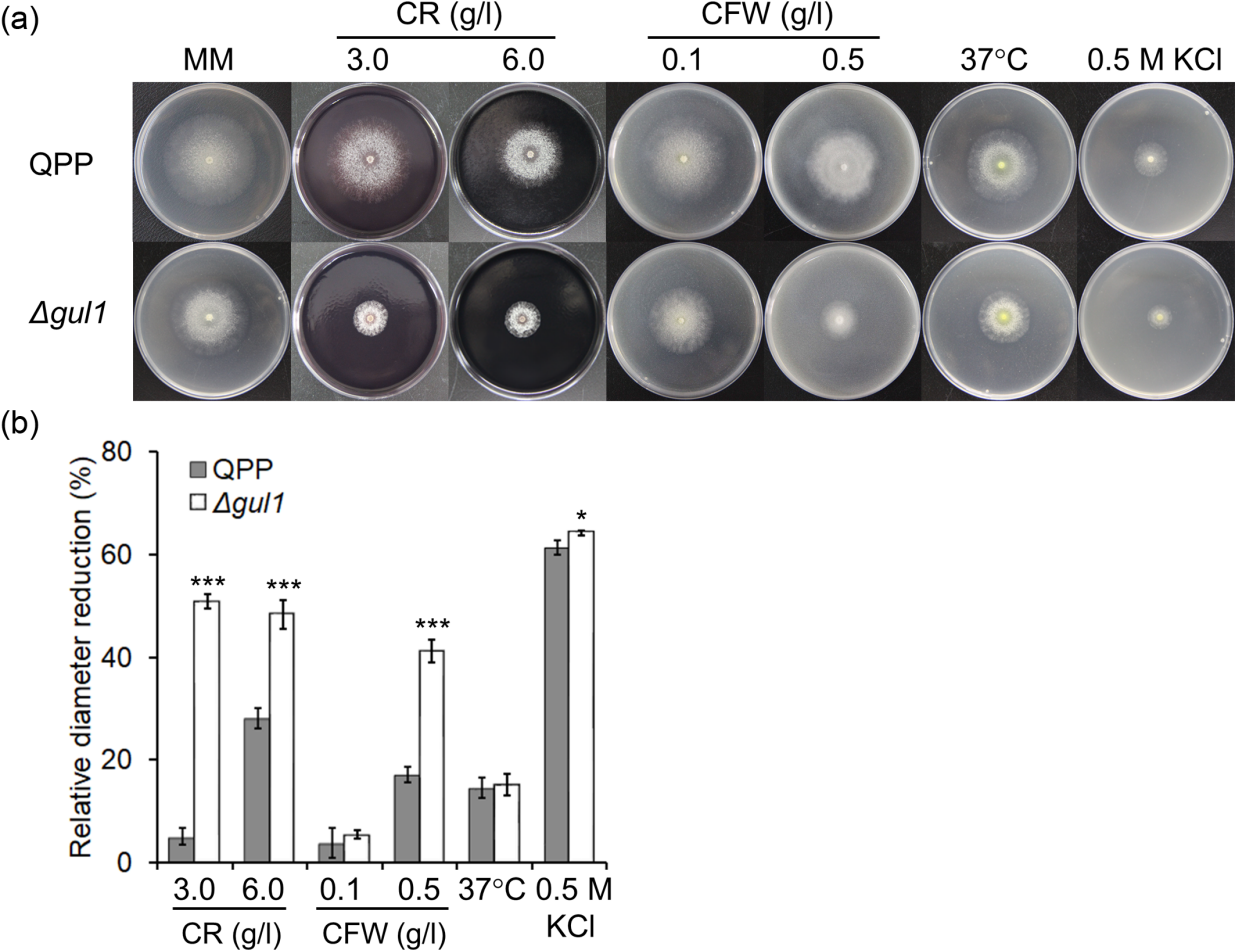
The sensitivity of Δ*gul1* and reference strain QPP to environmental stresses. (a) Colonies of strains under different cultivation conditions. (b) The reduction in colony diameter relative to that of the same strain growing on minimal medium at 30°C. Photos were taken and colony diameters were measured after 5 days of cultivation. MM, minimal medium. CR, Congo red; CFW, Calcofluor white. Data represent mean ± S.D. (error bars) from triplicate cultivations. ^*^p < .05; ^***^p < .001.

Both *S. cerevisiae SSD1* and *N. crassa gul-1* contribute to the maintenance of cell wall integrity (Lin et al., 2018; Mir et al., 2009). Given the similar effects of *gul1/gul-1* disruption on mycelial morphology between *T. reesei* and *N. crassa*, we examined the tolerance of Δ*gul1* to commonly used cell wall stressing dyes (Ram & Klis, 2006). Compared with the reference strain QPP, Δ*gul1* was significantly more sensitive to Congo red and Calcofluor white (Fig. 7), suggesting a weaker ability to modulate cell wall integrity in this mutant. The sensitivity to heat (cultivation at 37°C) and high osmolarity (0.5 M KCl) of Δ*gul1* was similar or slightly lower compared with QPP (Fig. 7), indicating that *gul1* is not involved in the tolerance to these stresses.

## Discussion

The morphology of mycelia is an important parameter in industrial fermentation for filamentous fungi (Cairns et al., 2019). For cellulase producing fungi, hyper-producing mutants with altered morphologies have been obtained through classical mutagenesis. For example, a mutant of *Myceliophthora thermophila* with high cellulase productivity formed small mycelial fragments in submerged culture, resulting in reduced viscosity (Visser et al., 2011). For *T. reesei*, a cellulase high producing mutant was found to form shorter, thicker and more frequently branched hyphae than the parent strain (He et al., 2016). Considering the close relationship between hyphal branch frequency, protein secretion ability and culture viscosity (Bocking et al., 1999), several genes involved in hyphal branching have been manipulated to test whether the production of secreted proteins could be improved. However, the mutants with increased hyphal branching (e.g., disruption mutants of the gene encoding Rho GTPase RacA/Rac1) did not always led to reduction in culture viscosity and/or enhanced protein secretion (Fiedler et al., 2018; Fitz et al., 2019). In this study, the disruption of gene *gul1* resulted in more branched hyphae and reduced culture viscosity in *T. reesei*, providing an effective target for rational strain engineering. It should be noted that the Δ*gul1* strain formed more lateral branches (Fig. 2a), while mutations in actin or RacA/Rac1 (regulating actin behaviors) genes usually formed more dichotomous branches (Kwon et al., 2011; Virag & Griffiths, 2004). Therefore, different types of “hyper-branching” might have different effects on mycelial morphology and protein secretion, which is worth being studied in the future.

The viscosity of fermentation broths during cellulase production showed rapid decreases between 24 and 48 hr, when cell biomass was still increasing (Figs 4 and 5). This inconsistence should be due to the change in mycelial morphology during fermentation, which needs to be further studied. In *A. niger*, the roughness of mycelial clumps was found to be correlated to broth rheology among several morphological parameters (Olsvik et al., 1993). Here, Δ*gul1* formed smaller clumps with less hyphal interactions (particularly in the early stage) compared with the reference strain, which could explain its lower broth viscosity throughout fermentation.

Many of the phenotypical changes observed in the Δ*gul1* strain (e.g., hyperbranching, lower viscosity and reduced cell wall integrity) are similar with those of *gul-1* deletion mutant in *N. crassa* (Lin et al., 2018). This highlights the significance of using the *N. crassa* gene deletion mutant library to identify genes determining important traits in biotechnology studies. To our knowledge, this is the first time that *gul1* manipulation was used for engineering the morphology of an industrial fungus. Comparative transcriptome analysis showed the transcript abundances of a set of genes with putative roles in cell wall remodeling or cell development changed in Δ*gul1* (Supplementary Table S2), of which many were also differentially expressed in the *N. crassa* Δ*gul*-*1 strain*. For examples, the genes TRIREDRAFT_22914 (encoding a β-1,3-glucanosyltransferase) and TRIREDRAFT_66792 (encoding a β-1,3-endoglucanase) were both downregulated in Δ*gul1*, and similar downregulations of their orthologs were also detected in *N. crassa* Δ*gul-1*. The orthologs of *Aspergillus nidulans phiA*, which is required for normal phialide development, showed significantly increased transcript abundances in both Δ*gul1* and Δ*gul-1*. The changes in the transcript abundance of these genes might contribute to the altered morphologies of *gul1*/*gul-1* disruption mutants.

Deletion of *gul-1* in *N. crassa* increased the production of extracellular β-glucosidase but not that of major cellulases (Lin et al., 2018). However, the *T. reesei* Δ*gul1* strain showed higher cellulase production level than the reference strain, which is beneficial from an industrial perspective. Interestingly, the cellulase genes were found to be downregulated in Δ*gul1* but not in Δ*gul-1*. Considering the rapid decrease of cellulase gene expression after early induction by cellulose (Cao et al., 2017), the effect of *gul1* disruption on cellulase gene expression needs to be investigated in time-course experiment.

The Cbk1-Ssd1 pathway is similar between *S. cerevisiae* and *N. crassa* in several aspects despite their different morphologies. In both species, the pathway is involved in the establishment of cell polarity and the maintenance of cell wall integrity. Recently, Gao et al. reported that the silencing of *cot-1* homolog in *T. reesei* resulted in more frequent hyphal branching and increased cellulase production in the early stage of fermentation (Gao et al., 2020). The results of them and us suggest that the above pathway is also conserved in *T. reesei*. While the molecular mechanism of regulation by Ssd1 in *S. cerevisiae* has been relatively clear (Kurischko, Kim, et al., 2011; Kurischko, Kuravi, et al., 2011), there is no direct evidence that its homologs also control the localization and translation of mRNAs encoding cell wall proteins. For the 14 Ssd1-bound mRNA targets supported by two independent studies (Hogan et al., 2008; Jansen et al., 2009), only *CTS1* (encoding an endochitinase) has an ortholog in *T. reesei* (*chi18-17*). Therefore, Ssd1/GUL1 might evolve with different targets in different fungal species. The identification of GUL1-bound targets (e.g., by RNA immunoaffinity purification) is expected to reveal the regulatory mechanism of GUL1, which is important for the understanding and precise engineering of cell morphogenesis in filamentous fungi.

## Supplementary Material

kuab012_Supplemental_Files

## Data Availability

The RNA-Seq data have been deposited in the Gene Expression Omnibus database under the accession number GSE147192.
