# Supplementary material for: Disruption of the Trichoderma reesei gul1 gene stimulates hyphal branching and reduces broth viscosity in cellulase production
Source: J Ind Microbiol Biotechnol. 2021 Feb 10;48(1-2):kuab012. doi: 10.1093/jimb/kuab012 (PMC9113457; doi:10.1093/jimb/kuab012)
Supplement: kuab012_Supplemental_Files [file kuab012_Supplemental_Files.zip › Supplementary file 1.pdf]

Disruption of the *Trichoderma reesei gul1* gene stimulates hyphal branching and reduces broth viscosity in cellulase production

Qinqin Zhao[1], Qin Liu[1], Qi Wang[2], Yuqi Qin[2], Yaohua Zhong[1], Liwei Gao[1,2]*, Guodong Liu[1,3]*, Yinbo Qu[1,3]

[1] State Key Laboratory of Microbial Technology, Shandong University, 27 Binhai Road, 266237 Qingdao, China

[2] Tobacco Research Institute of Chinese Academy of Agricultural Sciences, 11 Keyuanjingsi Road, 266101 Qingdao, China

[3] National Glycoengineering Research Center, Shandong University, 27 Binhai Road, 266237 Qingdao, China

*Corresponding authors. E-mail: lwgao@sdu.edu.cn (Gao L.) or gdliu@sdu.edu.cn (Liu G.); Tel.: +86 532 58632406; Fax: +86 532 58631501

Supplemental file 1:

Fig. S1; Fig. S2; Fig. S3; Supplementary Table S1.

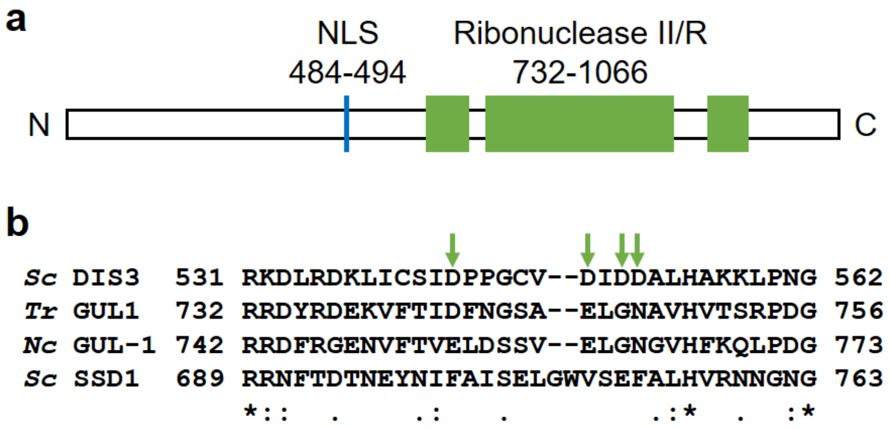

Supplementary Fig. S1 The sequence of *T. reesei* GUL1. **a** Schematic presentation of GUL1. The predicted domains (amino acids 625-700: IPR041505, Dis3-like cold-shock domain; 732-1066: IPR001900, Ribonuclease II/R; 1117-1189: IPR041093, DIS3-like exonuclease 2, C-terminal) are shown in green color. The nuclear localization signal (NLS) sequence was deduced from the alignment with *S. cerevisiae* SSD1 [1]. **b** Comparison of the amino acid sequence surrounding nuclease active site in RNase DIS3 of *S. cerevisiae* with the homologous regions in GUL1 orthologues. The aspartic acid residues involved in magnesium ion binding in DIS3 are marked with arrows [2]

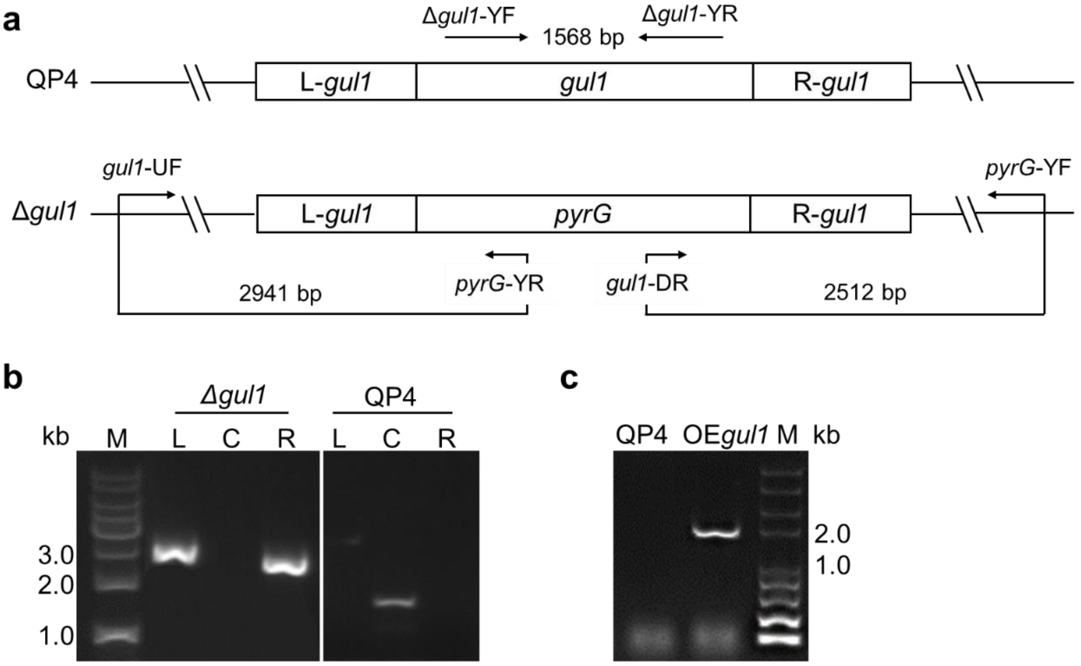

Supplementary Fig. S2 Identification of the *gul1* manipulation strains via PCR. **a** Schematic representation of the genomic organization of the *gul1* locus in QP4 and Δ*gul1* strain. **b** PCR analysis of *gul1* disruption strain and its parent QP4. Primer pairs gul1-UF/pyrG-YR (lane L) and pyrG-YF/gul1-DR (lane R) should generate a product of 2941 bp and 2512 bp in Δ*gul1*, respectively. Primer pair Δgul1-YF/Δgul1-YR (lane C) should generate a product of 1568 bp in QP4 but not in Δ*gul1*. **c** PCR analysis of *gul1* overexpression strain and its parent QP4. Primer pairs Ppdc1-YF/gul1-YR should generate a product of 2018 bp in OEgul1 but not in QP4

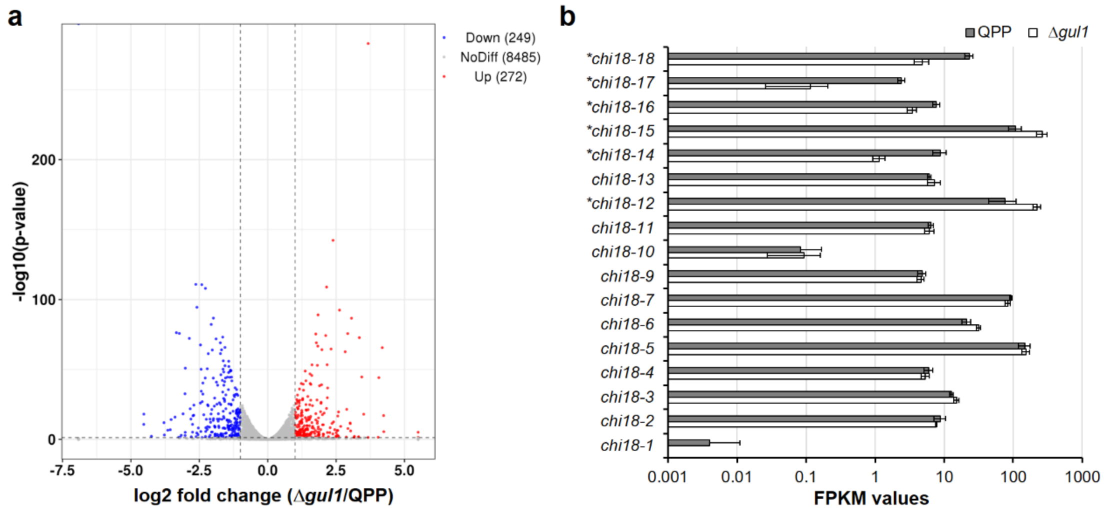

Supplementary Fig. S3 Transcriptional changes of genes between Δ*gul1* and reference strain QPP in cellulose medium. **a** Volcano plot of the transcriptional changes of all detected genes. **b** Transcript abundances (fragments per kilobase of exon model per million reads mapped, FPKM) of chitinase genes [3]. Data represent mean ± S.D. (error bars) from triplicate cultivations. Genes of significantly differential expression are labeled with asterisks

References

1. Kurischko C, Kuravi VK, Herbert CJ, Luca FC (2011) Nucleocytoplasmic shuttling of Ssd1 defines the destiny of its bound mRNAs. Molecular Microbiology 81:831-849. doi:10.1111/j.1365-2958.2011.07731.x
2. Lorentzen E, Basquin J, Tomecki R, Dziembowski A, Conti E (2008) Structure of the Active Subunit of the Yeast Exosome Core, Rrp44: Diverse Modes of Substrate Recruitment in the RNase II Nuclease Family. Molecular Cell 29:717-728. doi:10.1016/j.molcel.2008.02.018
3. Seidl V, Huemer B, Seiboth B, Kubicek CP (2005) A complete survey of *Trichoderma chitinases* reveals three distinct subgroups of family 18 chitinases. FEBS J 272:5923-5939. doi:10.1111/j.1742-4658.2005.04994.x

Supplementary Table S1 Primers used in this study

**Primer**	**Sequence**	**Product/Function**
gul1-UF	GCGGCAACTGAAGCATCAAC	*gul1* upstream sequence
gul1-UR	ATTGGGTGTTACGGAGCATTAGCCTG GTTCTCCTCCTCTGG	
gul1-DF	GATAACATTGACCAGCGGCTAGACAT CAAATAGTTGCCTTACC	*gul1* downstream sequence
gul1-DR	CCGTCCATAGTCCGAAAC	
pyrG-F1	CCAGAGGAGGAGAACCAGGC	*A. niger pyrG*
pyrG-R1	AGCCGCTGGTCAATGTTATC	
gul1-NF	GGCTGGGTTGAAGCTGGTTC	*gul1* knock-out cassette
gul1-NR	ACGAGGCTGTTTGGGTGAGA	
pyrG-YF	TGCAGGCTGCGCAACAGTAT	Δ*gul1* strain identification with gul1-DR
pyrG-YR	TGTAGCAGTGACTGACCCTT	Δ*gul1* strain identification with gul1-UF
Δgul1-YF	ACCCAAGGTCAATCTCAGGACC	*gul1* internal sequence
Δgul1-YR	TTTGCTCTCTCGTGCCAGGCGA	
Ppdc1-F	GCTCTTCTCCAAGCTGTACCCGT	P*pdc1* promoter
Ppdc1-R-gul1	GGCGGCTGCTGTTGCTGGTCCATGAT TGTGCTGTAGCTGCGCTGCT	
gul1-F-Ppdc1	AGCAGCGCAGCTACAGCACAATCAT GGACCAGCAACAGCAGCCGCC	*gul1* coding and downstream sequences
gul1-R-pyrG	AGACGAGACACGACGGCGGAATTCA ATTTGCTCATAATCTGCTCCGA	
pyrG-F-gul1	TCGGAGCAGATTATGAGCAAATTGA ATTCCGCCGTCGTGTCTCGTCT	*A. niger pyrG*
pyrG-R	CTAGCTTATCGATGGAACCAATG	
Ppdc1-NF	AGGACTTCCAGGGCTACTTGGCG	*gul1* over-expression cassette
pyrG-NR	TGTACTAGTTAGTAATGATGATG	
Ppdc1-YF	TGTTACGATGGATAGGTTCCAAC	OE*gul1* strain identification
gul1-YR	TTGCAGTGCCTGAAGCTGGCTGA	
